# QEPro: An ability measure of emotional intelligence for managers in a French cultural environment

**DOI:** 10.1007/s12144-021-01715-6

**Published:** 2021-04-23

**Authors:** Christophe Haag, Lisa Bellinghausen, Mariya Jilinskaya-Pandey

**Affiliations:** 1 Emlyon Business School, 23 avenue Guy de Collongue, 69130 Écully, France; 2 O.P. Jindal Global University, Narela Road, Sonipat, Haryana 131001 India

**Keywords:** Emotional intelligence, Psychometric testing, Management, Training & development

## Abstract

Managers’ interest in the concept of emotional intelligence (EI) has grown steadily due to an accumulation of published articles and books touting EI’s benefits. For over thirty years, many researchers have used or designed tools for measuring EI, most of which raise important psychometric, cultural and contextual issues. The aim of this article is to address some of the main limitations observed in previous studies of EI. By developing and validating QEPro we propose a new performance-based measure of EI based on a modified version of Mayer and Salovey’s (1997) four-branch model. QEPro is an ability EI measure specifically dedicated to managers and business executives in a French cultural environment (*N* = 1035 managers and executives). In order to increase both the ecological and the face validity of the test for the target population we used the Situational Judgment Tests framework and a theory-based item development and scoring approach. For all items, correct and incorrect response options were developed using established theories from the emotion and management fields. Our study showed that QEPro has good psychometric qualities such as high measurement precision and internal consistency, an appropriate level of difficulty and a clear factorial structure. The tool also correlates in meaningful and theoretically congruent ways with general intelligence, Trait EI measures, the Big Five factors of personality, and the Affect measures used in this study. For all these reasons, QEPro is a promising tool for studying the role of EI competencies in managerial outcomes.

For the past thirty years, an “affective revolution” (Barsade et al., 2003) has taken place in the workplace leading scholars around the world to study emotional intelligence (EI) in management. The intelligent use of emotions emerges as the new challenge for managers whose role is emotionally demanding (Ashkanasy et al., 2019; Ashkanasy & Daus, 2002; Caruso & Salovey, 2004; Côté, 2017; Haag and Getz, 2016, b; Humphrey et al., 2016), even more today with the COVID-19 crisis (Brooks et al., 2020). This worldwide health crisis - which is as much an economic and psychological crisis - has forced managers to be even more aware of their own emotions and the emotions of their subordinates, many of whom are anxious and struggle to regulate their stress at work (Serafini et al., 2020). Managers with a high level of EI have proved to be more able to regulate disturbing emotions for self and others (Caruso & Salovey, 2004; Farh, Seo, & Tesluk, 2012; Haag and Getz, 2016, b; Tse, Troth, Ashkanasy, & Collins, 2018) compared to those lacking such competence. Indeed, managers with a low level of EI, will need to develop their ability in order to successfully adapt - and help their team adapt - to this tense environment (Côté, 2017; McNulty & Marcus, 2020; Reiman et al., 2015; Schlegel & Mortillaro, 2019).

As such, the development of accurate EI measures and development programs dedicated to managers, especially those lacking EI, becomes crucial for organizations. Therefore, the aim of this article is to propose and validate an ability-based measure of EI with theory-based scoring, dedicated to managers.

## Defining EI

Two approaches of EI coexist today: (a) EI as an “ability” (one form of intelligence among others; Caruso & Salovey, 2004; Farh et al., 2012; Haag and Getz, 2016, b; Tse et al., 2018) and (b) EI as a “personality trait” (a trait among others; Bar-On, 1997; Goleman, 1995; Petrides & Furnham, 2000, 2003). Despite their differences, these two approaches of EI - each with their own merits (Mikolajczak, 2010) - converge on at least two aspects. First, EI has a positive impact on various factors, such as health, performance in the workplace, and the level of well-being (Mikolajczak, 2010; Zeidner et al., 2012b, a). Second, numerous studies have shown that these benefits are of interest to companies and their stakeholders (Côté, 2014).

“Personality trait” EI models depict a constellation of “traits”. These models integrate aspects of personality, motivation, affective disposition and intelligence into the EI approach (Matthews et al., 2004; Roberts et al., 2008; Zeidner et al., 2004). Trait models are strongly correlated to personality (Ciarrochi et al., 2001; Roberts et al., 2008), but some researchers argue that “trait EI explains additional variance over and above related traits such as alexithymia or the Big Five” to predict different outcomes such as work performance (Mikolajczak et al., 2010, p. 26). Despite the argument regarding Trait EI models having neurobiological correlates (Mikolajczak & Luminet, 2008), they might not measure anything fundamentally different from the Big Five (Davis & Humphrey, 2014) and therefore would not constitute a new form of intelligence (Matthews et al., 2004; Zeidner et al., 2004).

In contrast, the “ability” approach considers emotional intelligence to be a set of cognitive skills. The underlying idea is that emotions are information that are captured and processed by human brain (John D. Mayer et al., 2004). Ability approach of EI proposes a unique concept as it differs from analytical intelligence (Mayer et al., 2000a, b) and personality (Lopes et al., 2003). This EI model combines key ideas from the fields of emotion and intelligence (Mayer et al., 2000a, b). The arguments mentioned above led most researchers in the field of EI to consider the ability approach as the most promising one (Matthews et al., 2004; Schlegel & Mortillaro, 2019; Zeidner et al., 2004). This approach historically refers to Mayer & Salovey’s (1997) four-branch model presented below:

1. *Branch 1 - Perception, Appraisal and Expression of Emotion:* defined as the ability to accurately recognize emotions in self and others, to express emotions accurately and to discriminate between accurate and inaccurate / honest and dishonest expressions of feelings.
2. *Branch 2 - Emotional Facilitation of Thinking:* defined as the ability to orient attention to important information that helps in judgment and memory. This ability also encourages the change of individual perspective and supports the capacity to consider multiple point of views thanks to emotional mood swings.
3. *Branch 3 - Understanding and Analyzing Emotions:* defined as the ability to label emotions and recognize relationships among words and emotions themselves, to interpret the meaning of emotions, to understand complex feelings and to recognize transitions among emotions.
4. *Branch 4 - Reflective Regulation of Emotions to Promote Emotional and Intellectual Growth:* defined as the ability to stay open to feelings, to reflectively engage or detach from emotions, to monitor and to manage emotions in relation to oneself and others.

### Relevance of Ability EI for Managers

Three out of the four Mayer and Salovey’s branches (1, 3 and 4) have shown to be useful for managers (Schlegel & Mortillaro, 2019). For example, some studies have revealed benefits of a well-developed ability to recognize emotions (Branch 1) for various management related tasks. This ability is associated with various outcomes such as receiving better ratings from subordinates (Brotheridge & Lee, 2008; Byron, 2008) improving effectiveness in negotiation (Elfenbein et al., 2007) emerging as a leader in the group (Walter et al., 2012) and behaving as a transformational leader (Rubin et al., 2005).

*Researchers* have also shown that decision-makers who are able to understand emotions (Branch 3) take more efficient decisions as they are less influenced by irrelevant feelings - such as incidental anxiety - which are unrelated to the decision at hand (Yip & Côté, 2013). More recently, these researchers have observed that decision makers with low EI tend to adopt maladaptive decision-making, due to their incorrect appraisal of intensity of physiological arousal (Yip et al., 2020).

Finally, series of studies have shown that managers who display a strong ability to regulate their own emotions (Branch 4) tend to have a positive influence on their team’s performance (Lopes et al., 2006; Rice, 1999), experience an improved quality of their social interactions with others (Lopes et al., 2004, 2005), and, overall, feel more comfortable with themselves (Côté, 2010; Haag & Getz, 2016). Beyond the ability to regulate their own emotions, regulating the emotions of others (in particular those of their team members) is crucial for managers as these emotions are often important factors in team performance (Haag & Getz, 2016; Sy et al., 2005).

### Measurement of Ability-Based Model of EI

Researchers acknowledge that the best measurements of emotional competencies within the EI ability framework are performance-based tests (Petrides et al., 2007; Roberts et al., 2010). These types of tests are often opposed to self-report measures mostly used to measure “Trait EI”. Self-report questionnaires are subject to key limitations such as social desirability (Day & Carroll, 2004; Matthews et al., 2004), lack of respondent’s accuracy at estimating his/her own abilities (Brackett et al., 2006; Kruger & Dunning, 1999; Sheldon et al., 2014). In addition, “Trait EI” measures “violate the first law of intelligence” (Schlegel & Mortillaro, 2019, p. 560) because of their correlations with personality measures (Matthews et al., 2004, p. 225) and their lack of correlation with cognitive intelligence (Furnham & Petrides, 2003).

In contrast, performance-based tests of EI measure individual performance in solving emotional problems and performing emotional tasks. There is only one correct answer, others being incorrect. These tests “measure maximum performance in that individuals know that they will be evaluated, are instructed to maximize their efforts, and are measured over a short period of time” (Schlegel & Mortillaro, 2019, p. 560). However, there are psychometric issues and limitations to current performance-based EI tests (Fiori & Antonakis, 2011; Roberts et al., 2010).

#### Limits to Current Performance-Based EI Measures

Two main criticisms are often addressed to Ability EI measures: psychometric issues and lack of workplace relevance of the items (Davies et al., 1998; Schlegel & Mortillaro, 2019).

#### Psychometric Issues: Critics and Perspectives on Scoring of EI Ability Measures

Several researchers have highlighted psychometric issues with ability measures of EI mainly consensus, expert based scoring and their reliability and validity (Matthews & Zeidner, 2004; Maul, 2012; O’Connor et al., 2019).

Performance tasks involve right and wrong answers. However, determining the “right answer” for emotional intelligence tests is not as obvious as for other ability measures. Unlike conventional performance tasks, there is not a single answer to an item. Thus, “correct” answers have to be determined in different ways. Different methods have been identified (MacCann et al., 2004): researchers have set up a multi scoring method, thus contrasting with the scoring method used in traditional intelligence tests. Most frequently used methods to establish individual scores are “expert” scoring method and “consensus” scoring. The MSCEIT (John D. Mayer, 2002) and the STEU (MacCann & Roberts, 2008) both use these scoring methods.

The consensus criterion is the most widely used scoring principle (MacCann et al., 2004; John D. Mayer et al., 1999). It consists in taking the modal score for an item as the best (correct) response to the item. Distribution of scores of the experimental group is used to constitute the percentage grid to determine the attribution of individual scores. To properly discriminate scores, proportions of responses for each response modality are taken into account. Individual responses are coded into frequencies of responses given for each item by the entire sample. This method is often criticized because it assimilates the majority’s opinion to the right answer (Schlegel & Mortillaro, 2019). Consensus can help assess whether a response is incorrect, but it lacks the power to discriminate sensitive issues raised by social relationships. Thus, this method does not discriminate participants satisfyingly and a tendency towards right-asymmetric distributions is often observed (MacCann et al., 2004; Roberts et al., 2001).

With the expert criterion scoring method, experts first complete the questionnaire as participants. Once the responses are collected, the experts’ scores are transformed into frequencies. Then, participants’ scores are calibrated according to experts’ response frequencies (MacCann et al., 2004). It should be noted that expert scoring methods also present limits (Conte, 2005; Maul, 2012; O’Connor et al., 2019; Roberts et al., 2001) especially for abstract dimensions as regulation of emotions (Matthews et al., 2004).

In sum, these two methods both have strong limitations and despite their differences produce comparable results (MacCann et al., 2004; John D. Mayer et al., 1999).

New perspectives on scoring of EI ability measures have emerged in order to address those limitations such as theory-based item development and scoring. In this approach theories are used to define the characteristics representing high and low ability levels for each measured competence. These characteristics are then included as response options for each test items. The response option containing the characteristics associated with high ability levels “as defined by a given established theory, is specified as the objectively correct response and is expected to be chosen with a higher probability by individuals with a higher ability EI level” (Schlegel & Mortillaro, 2019, p. 561). For example, GECo (Schlegel & Mortillaro, 2019) and STEM (MacCann & Roberts, 2008) are based on this scoring method.

To anchor QEPro in the theory based-item development framework, each dimension was defined theoretically and then operationalized in reference to Management and Affective Sciences. Correct answers as well as distractors were created based on findings from experimental studies in the field of emotion and emotional regulation. Those studies combine findings from general as well as management-specific contexts. Following Ashkanasy and Daus’s (2005) classification of EI measures, QEPro thus belongs to the category of ability measures based on Mayer and Salovey’s model: maximum performance test with correct and incorrect answers. As such, all QEPro items are scored by standards based on emotion research / theory-based scoring rather than by consensus or expert rating (Kasten & Freund, 2015; MacCann et al., 2011).

#### Workplace Relevance of the Items: Situation Judgment Tests (SJTs)

A typical situational judgment test consists of a series of scenarios with a set of multiple-choice answers. SJTs are measurement methods that present applicants with job-related situations and possible responses to these situations. Applicants have to indicate which response alternative they would choose in real life. Therefore, the strengths of SJTs are that they show criterion-related validity and incremental validity above cognitive ability and personality tests (Lievens et al., 2008).

For example, Situational Test of Emotional Understanding (STEU, MacCann & Roberts, 2008) uses SJTs framework, but with scenarios unrelated to work. This test evaluates only one subdimension of EI, which is *Understanding Emotions*.

QEPro was conceived in the SJTs Framework. As such, common emotional situations encountered in managerial practices were collected from different occupational settings (pharmaceutical organizations, banking and finance, HRM...). This exploration of emotional situations led to the construction of vignettes (descriptions of situations familiar to managers) that are likely to occur in different managerial contexts.

In this framework, the ecological validity of the assessment (i.e. the predictive relationship between one’s performance on a set of tasks and one’s actual behavior in a variety of real-world settings) is improved thanks to the increased verisimilitude of the test items and real-world situations (Franzen & Wilhelm, 1996). Indeed, as the verisimilitude of the items refers to the “the similarity between the task demands of the test and the demands imposed in the everyday environment” (Spooner & Pachana, 2006, p. 328), the SJT anchors the vignette’s context in the managerial environment where they usually appear.

Similarly, the face validity of a test is defined by Streiner et al. (2015) as a subjective judgment, made by the target population, whether on the face of it, the instrument appears to be assessing the desired qualities. Although face validity is additional to more important psychometric qualities (such as criterion-related, content or construct validity) for psychological or educational tools intended for practical use, it is an important psychometric quality (Anastasi & Urbina, 1996; Brown, 1983; Cronbach, 1970). Indeed, a test with high face validity may have a better chance of inducing cooperation, reducing dissatisfaction and feeling of injustice among low scorers as well as increasing motivation during the test taking process (Nevo, 1985).

SJT framework and construction of QEPro items with a theory-driven scoring approach will increase both the ecological validity (MacCann & Roberts, 2008; Orchard et al., 2009) and the face validity of the test for the target population (Holden, 2010).

### Toward a New Measure of EI for Managers

Mayer and Salovey’s (1997) model was used as a framework for the construction of QEPro. We selected the three most robust branches of their model: *Emotion Identification, Emotion Understanding* and *Emotion Management* branches. We excluded the *Emotional Facilitation* branch because it did not load as a distinct factor in factor analyses and structural models (Ciarrochi et al., 2000; MacCann et al., 2014; Palmer et al., 2005; Schlegel & Mortillaro, 2019). The three selected dimensions were conceived at an ability level, as such, they were operationalized through a second level of emotional competencies which can be observed at the manager’s behavioral level. The following section describes QEPro’s three abilities with their corresponding emotional competencies (Table 1).

**Table 1:** Comparison of the Mayer & Salovey’s (1997) four-branch model and the QEPro Model of EI

Mayer & Salovey’s (1997) four-branch model	QEPro Model of EI
*Branch 1: Perception, Appraisal and Expression of Emotion*	1. Identifying Emotions (IE) **a) Scanning Physiological Manifestations** **b) Interpreting Emotional Cues.** **c) Identifying Emotional Triggers.**
*Branch 2: Emotional Facilitation of Thinking*	Excluded in QEPro Model
*Branch 3: Understanding and Analyzing Emotions; Employing Emotional Knowledge*	***2. Understanding Emotions (UE)*** **a) Understanding Emotional Timelines.** **b) Anticipating Emotional Outcomes.**
*Branch 4: Reflective Regulation of Emotions to Promote Emotional and Intellectual Growth*	***3. Strategic Management of Emotions (SME)*** **a) Selecting the Target Emotional State.** **b) Emotion Regulation.**

#### Identifying Emotions (IE)

IE refers to the ability to accurately identify emotions in self and in others. To identify emotions, one has to be able to gather, combine and process different types of emotional information: facial, postural and physiological cues, behavioral and cognitive manifestations, and triggers of emotions (e.g. Ekman, 1994; Ekman & Friesen, 1978; Scherer, 2000).

1. **Scanning Physiological Manifestations.** This competence refers to an individual’s ability to identify her/his own emotions according to an introspective analysis of the physical sensations experienced. Nummenmaa et al. (2014) mapped bodily sensations associated with different kinds of emotions. Each emotion was associated with a unique map. Scanning one’s body can therefore help recognize the type of emotion currently being experienced. Emotions can activate specific parts of the body (e.g. increased heat, tensed muscles) or deactivate them (e.g. numbness), accelerate rhythms of the body (e.g. heart rate, respiration) or slow them down (e.g. decreased heart rate) (James, 1922; Levenson, 2003; Nummenmaa et al., 2014). Noticing these changes and associating them with specific emotions creates a valuable source of information for better understanding and decoding one’s environment (Damasio, 1996). Similarly, this competence also serves to identify emotions of others. The physiological components of emotional responses of others can be both observed (e.g. accelerated heart rate, breathing, muscle tension…) as well as experienced in self through an emotional contagion process (Hatfield et al., 1993).
2. **Interpreting Emotional Cues.** In addition to the physiological level, emotions can also be identified through their cognitive manifestations; behavioral action tendencies; vocal, postural and facial cues; and the associated subjective-experiential component (Frijda, 1986; Luminet, 2008; Scherer, 1984). These cues differ in intensity and are not always easy to recognize in self or others. For instance, the cue may consist of only weak signals, such as a slight smirk, a furtive look, or a slightly raised eyebrow.
3. **Identifying Emotional Triggers.** In addition to their manifestations, emotions are also associated with specific triggers (Basch & Fisher, 1998; Matthews et al., 2004). Those triggers indicate that something occurred in one’s environment (e.g. danger, a loss, etc.), which can have a positive or negative impact on self and others. Therefore, this third dimension refers to the competence of individuals to identify the specific triggers of their own emotional state and that of others. Identifying the causes of emotions is important as it allows to complete the identification process and initiate the understanding of what to do in order to better adapt to a situation (Matthews et al., 2004).

The ability to use those three competencies in a concomitant and combined manner increases the accuracy and the efficiency of the identification process. As such, the ability to identify emotions can be seen as a meta-competence.

### Understanding Emotions (UE)

UE refers to the ability to accurately appreciate the intensity level of a given emotional state and to anticipate its evolution over time and its consequences on self and others (John D. Mayer & Salovey, 1997; Schlegel & Mortillaro, 2019).

1. **Understanding Emotional Timelines.** This competence allows an individual to assess the intensity of her/his emotional state (and that of others) and to anticipate its evolution over time. Emotions of the same category will logically follow one another along an intensity continuum over time (e.g. before feeling anger or becoming enraged, an individual will experience different, less intense emotional states; Plutchik, 1984). Knowing this sequencing not only allows one to estimate the precise intensity level currently being experienced, but also to make predictions regarding likely future emotional states, for both self and others.
2. **Anticipating Emotional Outcomes.** This competence allows an individual to anticipate the positive and negative consequences of an emotion. Each emotion can be associated with a specific adaptive role (Caruso & Salovey, 2004; Damasio, 1994; Darwin, 1872; Fredrickson, 2002). The outcomes of emotions (e.g. behaviors, action tendencies, cognitive patterns…) are associated with both positive and negative implications (Tran, 2007). For example, anger, on the one hand, can enhance aggressiveness which could nurture conflict and potential fighting while, on the other hand, anger can also help to gain self-confidence and the right amount of energy to achieve one’s goal (Tran, 2007). As such, any emotion, be it pleasant or unpleasant, has implications on the self, others, and groups. These consequences are both negative and positive and thus can facilitate (or hamper) one’s performance in a given situation.

These competencies are essential in order to help prepare an efficient and strategic management of emotions to adapt to a situation.

### Strategic Management of Emotions (SME)

SME refers to the ability to reflectively manage emotions, to influence emotions one experiences, to choose when to experience them and how to feel and express them, which is in line with Gross’ (2013) model of *Emotion Regulation.* In QEPro’s model, this ability is operationalized with two emotional competencies: the competence to define an emotional goal or target emotional state (Gross, 2013; Mikolajczak & Desseilles, 2012) and the competence to implement emotional regulation strategies in order to achieve the emotional goal (Gross, 2013).

1. **Selecting the Target Emotional State.** This competence enables individuals to choose the target emotional state that best fits the situation in order to enhance performance and well-being for self and others. In order to select the right target emotional state, individuals have to consider three parameters (Gross & Thompson, 2007; Mikolajczak et al., 2014) (a) Duration of the emotion: they can choose to extend or shorten their current emotional state (or that of others), (b) level of intensity: they can increase or decrease the level of intensity of their current emotional state (and that of others), (c) nature of the emotion: they can choose to switch to a different emotional state that better fits the situation. In addition, our clinical experience led us to add a fourth parameter (d) complementary emotion(s). An individual can also choose to activate one or a combination of additional emotions, emotions that are complementary to the experienced emotional state. These emotions, so-named resource emotions, are selected based on their potential positive outcomes in a given situation.
2. **Emotion Regulation.** This competence enables to choose the best possible emotional regulation strategy to achieve a given target emotional state. Gross (1999) defines two types of regulation strategies: antecedent-focused strategies (selection or modification of the situation; attention deployment; cognitive reappraisal) and response-focused strategies (modulation of the emotional response). These emotion regulation strategies can be used in order to influence one’s own emotion or that of others.

SME involves the ability to combine those two competencies in order to adapt efficiently to the workplace and enhance well-being.

## Method

Test development and validation is a continuous process of collecting evidence related to reliability and validity of test scores according to different criteria (e.g. convergent and divergent measures related to ability EI; Nunnally, 1994) and thereby improving quality of items (Downing, 2006) in order to reach sufficient standards for psychometric ability-based measurement. This main objective is further divided into sub-objectives related to different stages of test development and validation. First, we present a summary of the test development process. Then, we present the dimensions of the final version of QEPro. Furthermore, we describe the participants and procedure as well as the material and measures used in the validation study.

### Development of QEPro

The test was developed within a Multiple-Choice-Questions with Single correct Answer (MCQ-SA) framework in an online format. MCQ-SA format was selected due to its ease of administration and better measurement properties over other formats (Bible et al., 2008; Simkin et al., 2011). For each of the items - in addition to the correct answer – 4 to 5 distractors were retained in the final version as recommended by literature in order to maximize psychometric qualities of the questionnaire (Dickes et al., 1994; Nunnally, 1994).

For the first version of the QEPro Questionnaire, the authors generated 8 to 15 theory-based items per dimension (total of 70 items) as it is advised to develop an initial pool of twice the final number of items (Dickes et al., 1994). Previous studies have demonstrated that systematic item review by experts has a positive impact on test validity (Downing & Haladyna, 1997). Thus, the first version of QEPro was submitted to a group of experts in the fields of management and emotions. Based on guidelines established by previous studies on expertise, experts were selected on the following criteria (a) presence of initial training in their field (Chi, 2006), (b) at least 10 years of experience in their field (Ericsson, 1999; Ericsson et al., 1993; Howe, 2001; Simon & Chase, 1973), (c) pursuing continuous education and training in their field of expertise (Ericsson et al., 1993), and (d) being recognized by peers and professional associations as an expert (Chi, 2006). Fulfilling all of the above criteria, 25 experts were identified (9 professional coaches and 16 senior managers). Their qualitative feedback was highly encouraging as the experts were able to identify the correct answer for every item in the first three subscales. Two items were discarded from the *Understanding Emotional Timelines* subscale and four, from the *Anticipating Emotional Outcomes* subscale due to wording of the items. The last two subscales required more adjustments. For the subscale *Selecting the Target Emotional State* five items, for which the experts could not identify the correct answer, were discarded. Finally, for the subscale *Emotion Regulation* one situation was discarded. Overall, the experts judged QEPro useful and of interest for improving management outcomes. The group highlighted the potential benefits of the tool for recruitment and the development of managers’ and executives’ emotional skills to promote well-being and performance at the workplace. This qualitative feedback lead to select 58 items for the quantitative pre-test (Table 2).

**Table 2:** Number of items per subscales for each of the three consecutive versions of QEPro

Subscales	First Version	Pre-test Version	Final Version
Identifying Emotions (IE)			
Scanning Physiological Manifestations	8	8	4
Interpreting Emotional Cues	8	8	5
Identifying Emotional Triggers	8	8	5
Understanding Emotions (UE)			
Understanding Emotional Timelines	10	8	6
Anticipating Emotional Outcomes	10	6	6
Strategic Management of Emotions (SME)			
Selecting the Target Emotional State	15	10	5
Emotion Regulation	11	10	5
Total	70	58	36

The quantitative pre-test aimed to assess the difficulty and the discriminant power of the 58 items. It was administered to a sample extracted from the target population, namely managers and executives. The sample was composed of 466 managers (284 men and 182 women) with an average age of 26.6 years (*SD* = 6.65). Most of the participants had significant managerial experience (25% and 67% of the participants had, respectively, between 3 to 10 years, and over 10 years of managerial experience).

Statistical item difficulty as well as discriminant analysis were conducted. Only the items meeting the following criteria were kept for the final version of QEPro (a) the discrimination index of the item was superior to .20 and inferior to .80, (b) no single distractor was chosen more often than the right answer, and (c) each distractor was selected as the right answer by some participants. This analysis led us to discard 22 items.

The final version was composed of 36 items organized in seven subscales (Table 2). QEPro was administered online using Qualtrics platform without limited time to complete the test. The subscales, along with an example of items, are detailed below.

### Final Version of QEPro

#### Identifying Emotions (IE)

##### Scanning Physiological Manifestations

This subtest measures the test-taker’s ability to accurately identify physiological changes associated with a specific emotional state. The subtest is composed of four items. The test taker has to associate a specific emotion with one of the three bodies presented in the item. Each of the bodies reflects a different emotional state as mapped by Nummenmaa et al. (2014).

E.g. “Among a set of three bodily maps of emotions each with distinct topographical bodily sensations, identify the bodily map corresponding to the emotion stated in the question.”

##### Interpreting Emotional Cues

This subtest measures the test taker’s competence to accurately identify an emotional state based on different emotional cues or manifestations as described by Scherer (1984). The subtest is composed of five items. Each item describes an emotion based on three to four emotional cues. The test takers’ task is to select which emotion (among the 6 options proposed) is described in the item.

E.g. “A pleasant warmth invades my face and my voice is characterized by great loudness, high pitch, and fast speed. I am straightened up and I want to celebrate this feeling with those around me.”

(a) Pride; (b) Joy; (c) Satisfaction; (d) Surprise; (e) Hope; (f) Awe.

##### Identifying Emotional Triggers

This subtest measures the test taker’s ability to associate a trigger with the corresponding emotion. The subtest is composed of five items. Each item describes an emotional trigger, the test-taker’s task is to select among the 6 answer options, the emotion corresponding to the described trigger.

E.g. “A situation where your boundaries are offended can lead to:”

(a) Disgust; (b) Fear; (c) Anger; (d) Guilt; (e) Envy; (f) Sadness.

#### Understanding Emotions (UE)

##### Understanding Emotional Timelines

This subtest measures the test taker’s ability to group emotion words per family and place them correctly on an emotional intensity continuum in line with Plutchik’s Wheel of Emotions (1984). This subtest is composed of six items. Each item presents an emotional intensity continuum graph (low intensity, medium intensity and high intensity). Two of the emotional words are missing and the test taker is asked to select the appropriate words to complete the graph from 6 answer options.

E.g. “Among the list presented below identify the word best corresponding to X and the word best corresponding to Y.

X (Low intensity) – Anger – Y (High Intensity)”.

(a) Terror; (b) Sorrow; (c) Gloom; (d) Annoyance; (e) Fear; (f) Rage.

##### Anticipating Emotional Outcomes

This subtest measures the test taker’s ability to identify possible positive and negative implications of emotions on self, others and the group. This subtest is composed of six items. Each item describes a situation with a positive or a negative outcome linked to a specific emotion. The test taker has to select among the 6 answer options the emotion which could lead to such outcomes.

E.g. “I strengthen the sense of belonging to the group, increase self-confidence, enhance and drive task engagement.”

(a) Joy; (b) Pride; (c) Interest; (d) Hope; (e) Satisfaction; (f) Relief.

#### Strategic Management of Emotions (SME)

##### Selecting the Target Emotional State

This subtest measures the test taker’s ability to select the most suitable and efficient emotional state for a goal in a given situation. This subtest is composed of five vignettes. These vignettes describe managerial situations that are likely to occur in organizations. In order to achieve the situational goal mentioned in the vignette, the test-takers are required to select the most suitable emotion to experience among six answer options. The correct answer in the following example is based on emotional recall and mood congruent memory’s studies (e.g. Gilligan & Bower, 1983).

E.g. “You have misplaced an important document. You misplaced it the other day while coming out of a meeting which was especially annoying. To increase your chances of finding the file quickly, what emotion should you activate in yourself?”

- Guilt; (b) Apprehension; (c) Joy; (d) Annoyance; (e) Pride; (f) Sadness

##### Emotion Regulation

This subtest measures the test taker’s ability to select the most efficient emotion regulation strategy to achieve a specific emotional goal. This subtest is composed of five vignettes. Each vignette describes a managerial situation along with an emotional goal. The test takers are asked to select the regulation strategy they would most likely use in real life to achieve the emotional goal mentioned in the vignette among five answer options. Each of the answer options were generated within the emotion regulation framework defined by Gross (1999) and corresponds to one of the emotional regulation strategies he identified (e.g. situation selection; cognitive reappraisal; modulation of the emotional response…). The correct answer was defined as the strategy which allows to attain the emotional goal mentioned in the vignette, at the right intensity level.

E.g. “Bob, one of your employees, who is rather shy and quiet, has exceeded his target despite a difficult context (lack of means and strong pressure). You want to motivate him further, and want to make him feel proud. Select the strategy which you would use in real life to make him feel proud?”

- You remind him that the team has been of an invaluable help to him in carrying out this project.
- You ask him to stand up and lift his chin up towards the sky.
- You congratulate him during the weekly meeting, in front of the whole department and ask his colleagues to applaud.
- You encourage him to list the targets he wants to achieve in the next semester.

## Participants and Procedure

A total of 1035 managers and business executives (535 men, 500 women) with 1 to 25+ years of experience in management participated in the validation study. The average age was 43.9 years (*SD* = 8.25). Three levels of management were identified: front line management (*N* = 400), middle management (*N* = 347), and top management (*N* = 288). Most of the managers held a graduate degree (*N* = 684).

The managers belonged to different divisions within their companies (Table 3). The most represented divisions were general management (20.5% of the sample), sales (18.2%), and human resources (11.7%).

**Table 3:** Frequency and Percentages of Managers per divisions in the QEPro validation study

Department	Frequency	Percentage
Sales	188	18.2
Accounting	9	0.9
Advice/Consulting	62	6
General Management	212	20.5
Law/Legal Services	12	1.2
Finance/Management Control	96	9.3
Training/Coaching	20	1.9
Logistics/Purchasing	45	4.3
Marketing/Communication	74	7.1
Human Resources	121	11.7
Information Systems	32	3.1
Other	164	15.8
Total	1035	100%

All participants were recruited via email through a variety of sources (alumni directory of a top French business school, online professional networking sites...) and voluntarily participated in the study without financial incentives and being aware of the confidentiality of their answers.

All participants completed QEPro (seven subscales) along with other tests and questionnaires online (via the Qualtrics software package). The constructs measured by all these tests can be categorized in three broad areas: Ability, Personality and Trait EI & Affective Measures (Table 4). The test administration extended over a period of six weeks in late 2016 and all participants took the tests in the same order.

**Table 4:** Assessment tools used in the validation study

Tool	Acronyme	Author	French Version
Ability Measure			
Advanced Progressive Matrices – Short Form	APM-SF	Arthur Jr and Day, 1994	
Personality and Trait EI Measures			
Big Five Inventory	BFI	John, & Robins, 1991	Plaisant et al., 2010
Trait Emotional Intelligence Questionnaire	TEIQUE	Petrides, 2009	Mikolajczak et al., 2007a
Emotional Intelligence Scale	EIS	Schutte et al., 1998	Rossier, unsubmitted
Affective Measures			
Toronto Alexithymia Scale	TAS-20	Bagby, Parker, & Taylor, 1994	Loas et al., 1996
Maslach Burn-Out Inventory	MBI-GS	Maslach, Jackson, & Leiter, 1996	Dion & Tessier, 1994
Basic Empathy Scale for Adults	BES-A	Jolliffe & Farrington, 2006	Carré et al., 2013
Consideration of Future Consequences Scale	CFC-14	Strathman et al., 1994	

### Materials and Measures

#### Ability Measure

*The Advanced Progressive Matrices – Short Form (APM-SF;* *Arthur Jr & Day,* 1994*)* measures general cognitive ability. This test is composed of 20 items to be solved in a maximum of 20 min. Each item consists of a matrix of nine boxes (3 × 3) one of which is left blank. The participants are required to choose the correct response among the eight alternatives presented below the matrix. The internal consistency and the test-retest reliability of the APM-SF are good (Cronbach’s *α* = .72; test-retest reliability *r* = .75).

#### Personality and Trait EI Measures

*The Big Five Inventory (BFI-FR;* *Plaisant* et al.*,* 2010*)* was used to measure personality. BFI-FR is a 45-item measure of personality. The five factors of personality measured are: extraversion (E = 8 items), agreeableness (A = 10 items), conscientiousness (C = 9 items), neuroticism (*N* = 8 items), and openness (O = 10 items). Each item was rated on a five-point Likert-type scale from “strongly disapprove” to “strongly approve.” The reliability of the scale is good (Cronbach’s *α*: E = .82; A = .75; C = .80; *N* = .82; and O = .74).

*The Trait Emotional Intelligence Questionnaire (TEIQue-SF;* *Mikolajczak et al.,* 2007a*,* b*,* c*).* This self-report measure is composed of 30-items rated on a 7-point Likert-type scale ranging from “Completely Disagree” to “Completely Agree”. The TEIQue-SF provides a general assessment of Trait EI. TEIQque-SF scales have good internal consistency (Cronbach’s *α* between .71 and .91).

*The Emotional Intelligence Scale (EIS;* *Schutte* et al.*,* 1998*).* This self-report measure is composed of 33 items (three of which are reversed) to be evaluated on a 5-point Likert-type scale ranging from “Strongly Disagree” to “Strongly Agree”. The EIS provides a general assessment of Trait Emotional Intelligence based on the earlier EI model of Salovey and Mayer (1990). The EIS has a good reliability (Cronbach’s *α* = .87; Stability at 2 weeks, *r* = .78).

#### Affective Measures

*The Toronto Alexithymia Scale (TAS-20;* *Loas* et al.*,* 1996*)* is a 20-item measure of Alexithymia. Alexithymia is a personality construct which reflects a significant deficit in experiencing, expressing and regulating emotions. The TAS-20 is composed of 20 items to be rated on a 5-point Likert-type scale. It consists of three factor scores measuring: difficulty in identifying one’s feelings (7 items), difficulty in describing one’s feelings (5 items), and externally-oriented thinking (8 items). The reliability of the scale is good (Cronbach’s *α* = .81; Test re-test at 3 weeks, *r* = .77). It has demonstrated convergent and discriminant validity, and scores show high agreement with observer ratings of alexithymia (Parker et al., 1993).

*The Maslach Burn-Out Inventory (MBI-GS;* *Dion & Tessier,* 1994*)* evaluates burnout in general terms (not specific to any particular profession). MBI-GS assesses the psychological impact of the emotional and affective demands of intense involvement and investment in one’s work. MBI-GS is composed of 16 items to be rated on a Likert-type scale ranging from “Never” to “Always”. The MBI-GS provides three scales: Exhaustion (5 items), Cynicism (5 items), and Loss of Professional Efficacy (6 items). The tool’s validity has been shown to be satisfactory (Aguayo et al., 2011; Langballe et al., 2006; Schutte et al., 1998).

*The Basic Empathy Scale for Adults (BES-A;* *Carré* et al.*,* 2013*) is* composed of 20 items to be rated on a 5-point Likert-type scale ranging from “Strongly Disagree” to” Strongly Agree.” The BES-A provides three scores: emotional contagion, cognitive empathy, and emotional disconnection. The factors are defined by Carré et al. (2013) as follows (a) *Emotional Contagion* refers to “a persons’ ability to automatically replicate another person’s emotion”, (b) *Cognitive Empathy* corresponds to “a persons’ ability to understand and to metalize another person’s emotions”, (c) *Emotional Disconnection* is defined as a “regulatory factor that involves self-protection against distress, pain, and extreme emotional impact” (Carré et al., 2013, p. 681). BES-A subscales have good reliability (Cronbach’s *α* between .69 and .82).

*The Consideration of Future Consequences Scale - French version (CFC-14;* *Strathman* et al.*,* 1994*)* measures the consideration of future consequences. This dimension is conceived as a stable trait describing, at one end, individuals who prefer to rely on immediate consequences or the satisfaction of immediate goals and, at the other end, those who prefer to defer the satisfaction of their immediate needs to take care of their overall well-being. The CFC-14 is composed of 14 items to be rated on a 5-point Likert-type scale ranging from “Extremely Uncharacteristic” to “Extremely Characteristic.” The reliability of the scale is good (Cronbach’s *α* between .80 and .86 depending on the sample; Stability at two weeks *r* = .76 and Stability at five weeks *r* = .72).

## Results

First, we examined the reliability of QEPro: (a) sensitivity and discriminating power of QEPro items, (b) structure of the questionnaire through confirmatory factor analysis at both the item level and the dimension level and finally, (c) stability of the questionnaire over time (test-retest reliability).

Second, we assessed the criterion validity using a Multi Trait - Multi Method analysis with reference to four categories of measures (a) Demographic Data, (b) Ability Measure, (c) Personality and Trait EI, and (d) Affect Measures.

### Reliability Study

#### Sensitivity and Discriminating Power

The initial step of this analysis enabled us to select items with a satisfactory discriminant power to use in the final version of the questionnaire. We used three criteria to judge the quality each item: Difficulty Index, Discrimination Index and quality of distractors (Dickes et al., 1994).

The Difficulty Index is the ratio of respondents who correctly answered the item to the total number of people in the sample. The index ranges from 0 to 1 where 0 indicates that no one identified the correct answer and 1 indicates that everyone identified the correct answer. The lower the index score for the item, the more difficult the item. To select the most suitable items for the test, we followed Crocker and Algina’s (1986) criteria. They suggested an acceptable range of .20 to .80 for a four-choice item if those intend to measure a range of ability levels. This analysis also helped us organize the items within each subscale: we placed the easiest items at the beginning of each subscale and the most difficult question at the end.

The Discrimination Index assesses the discriminating power of an item, i.e. the ability to distinguish individuals belonging to the high-performers group from those belonging to the low-performers group as clearly and precisely as possible. This index is defined as the difference between the proportion of correct answers to an item among the highest scoring individuals, and the proportion of correct answers to the item among lowest scoring individuals (Bond & Fox 2001). To calculate this index (D), we divided the sample into three groups based on their total score: the 27% highest scorers (high performers), the 27% lowest scorers (low performers) and the 46% with middle scores. Only the two extreme groups (high and low performers) were used to calculate the index. Within each group we calculated the proportion of success in answering the item correctly. This proportion will be referred to as RHigh and RLow for high- and low-performers groups respectively and represents the ratio between the number of individuals who successfully answered the items and the total number of individuals. A Discrimination Index (Eq. 1) was computed for all the items of the seven subscales.

1 $$ {\displaystyle \begin{array}{l}\mathrm{D}=\mathrm{RHigh}-\mathrm{RLow}\\ {}\ \mathrm{D}\mathrm{iscrimination}\ \mathrm{Index}\end{array}} $$

The minimum accepted level of item discrimination was set at .20 because it provides a satisfactory level of discrimination as suggested by Nunnally and Bernstein (1967). Before computing the discrimination index for an item, the item’s score was subtracted from the total score, thereby correcting for spuriousness.

The analysis of the distractors allowed to identify distractors that were not performing correctly, either because they are infrequently chosen (which would indicate that the distractor is too unlikely), or because conversely, they are chosen too often (which would indicate that the distractor is far too close to the correct answer for it to be considered incorrect). Each item was expected to have a roughly equal distribution of responses across all the distractors.

In order to represent the full spectrum of emotions experienced in the workplace we aimed to keep a balance between the number of items on pleasant and unpleasant emotions within each subscale.

The 36 items of QEPro’s final version presented satisfactory discriminant qualities (.20 < D < .80). The items were consistent and allowed for a precise positioning of individuals in different ability groups with distractors that were neither over-selected nor under-selected by respondents.

#### Structure of the Questionnaire

After studying the discriminating power of the items, we moved on to examine the structure of QEPro by using Confirmatory Factor Analysis. We examined the structure of the questionnaire at both the item and the dimension levels.

**Item Level.** In order to test that the seven-dimensional model can explain the relationship among the items, the structure of the questionnaire was studied using CFA. The items were postulated to load on one factor only with no cross-loadings, while the seven factors were postulated to be inter-correlated. Three covariances were added.

Within the *Scanning Physiological Manifestations* dimension, one covariance was added between the only two items dealing with emotions that elicit high activation (as opposed to a high deactivation) in the body.

Within the *Understanding Emotional Timelines* dimension, one covariance was added between the only two dimensions dealing with pleasant emotions.

**Fig. 1:**
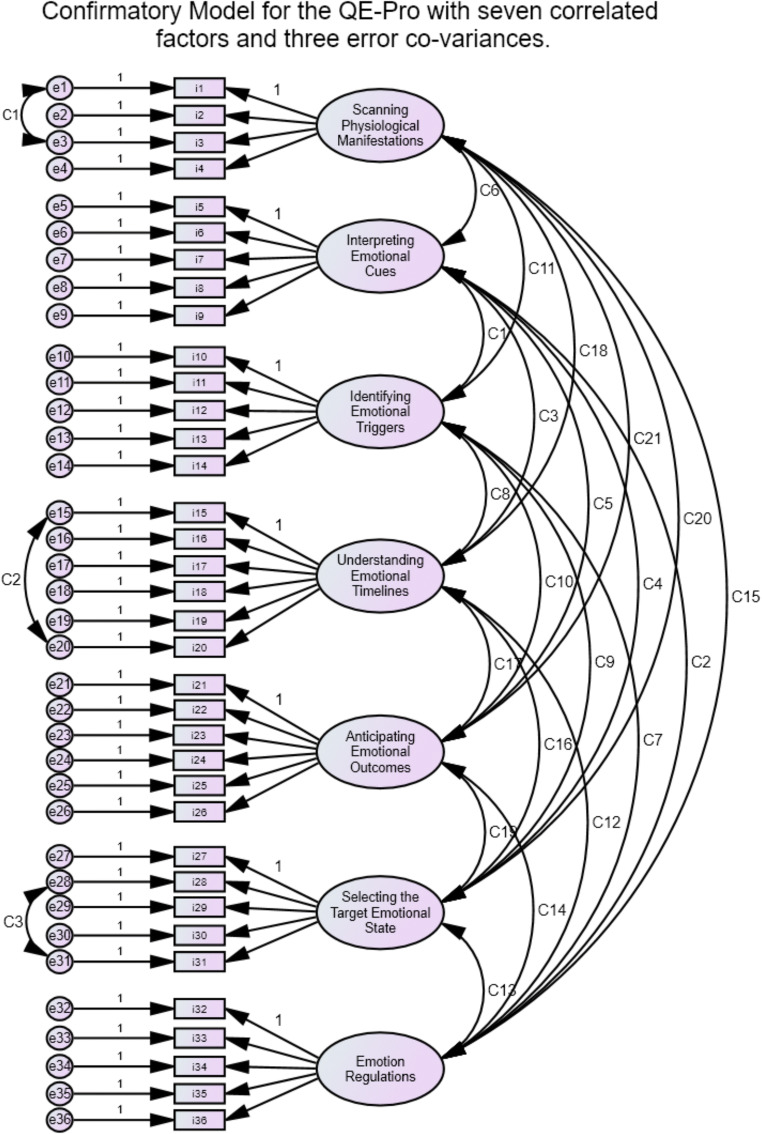
Confirmatory Model for the QE-Pro with seven correlated factors and three error co-variances

Within the *Selecting the Target Emotional State* dimension, one covariance was added between two items. Those two share the same strategic process (re-activating the same state in which an event occurred) as far as defining the target emotional state is concerned which differs from all the other items of the scale. The final model is presented in Fig. 1.

Recent studies and simulations (Glockner-Rist & Hoijtink, 2003; Savalei et al., 2015) have argued that using Robust Weighted Least Squares (WLSMV) as the extraction method (as opposed to Maximum Likelihood) is appropriate for dichotomous variables (L. K. Muthén & Muthén, 1998). This will estimate the appropriate matrix for the factor extraction, based on tetrachoric correlations (Barendse et al., 2015; Flora & Curran, 2004).

MPLUS (Version 6.12. [Computer Software]. Los Angeles, CA: Muthén & Muthén) was used to estimate the parameters of the model. Although the test of exact fit proved to be significant (χ^2^ = 631.413, *df* = 536), the test of close fit (RMSEA = .013) is inferior to the minimum of .05 required to prove an acceptable adjustment (Browne & Cudeck, 1993). This hypothesis can be accepted at a high probability threshold (p RMSEA <= .05 > .99). In his “Guidelines Concerning the Modelling of Traits and Abilities in Test Construction”, Schweizer (2010) advises on the reporting of CFI (Comparative Fit Index; Bentler, 1990) and SRMR (Standardized Root Mean Square Residual) in addition to the χ^2^ and the RMSEA. As our data are categorical, the WRMSR (Weighted Root Mean Square Residual) will be reported instead of the SRMR (DiStefano et al., 2018; Flora & Curran, 2004; B. Muthén, 1978).

The Comparative Fit Index is acceptable (CFI = 0.958) and is superior to the .95 limit (Hu & Bentler, 1999). The WRMSR is acceptable as it is inferior to the cut-off score of 1 (WRMSR = .978; DiStefano et al., 2018).

##### Dimension Level

A CFA conducted with MPLUS (Version 6.12 [Computer Software]. Los Angeles, CA: Muthén & Muthén) confirmed that the correlated multi-dimensional structure of QEPro presents a better fit than a hierarchical structure with a general second order factor saturating the seven first-order factors (one for each dimension of QEPro).

**Fig. 2:**
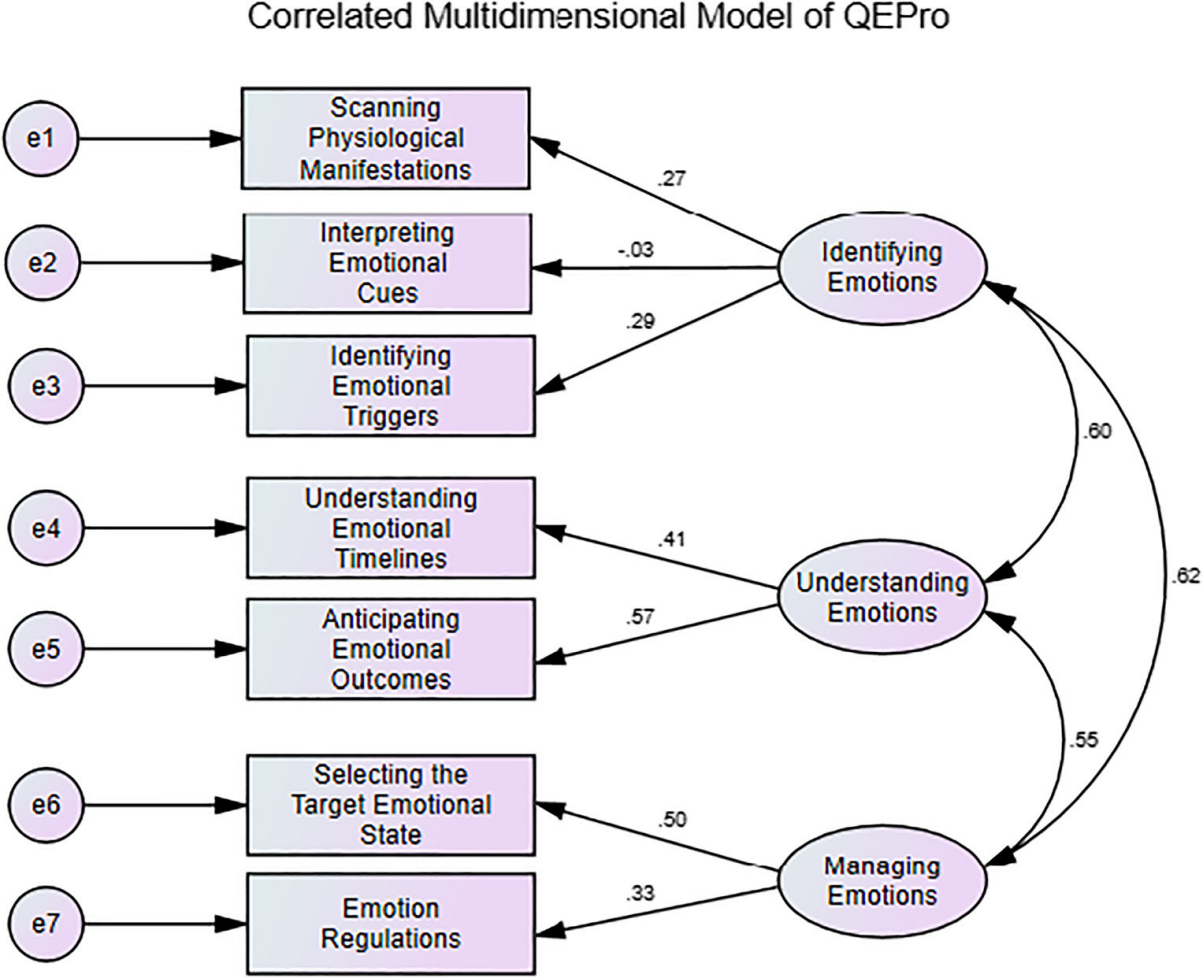
Correlated Multidimensional Model for the QE-Pro with three correlated second-order dimensions

Indeed, QEPro measurement model indicates a good fit of the data to the correlated multidimensional model (Barrett, 2007; Browne & Cudeck, 1993; Hu & Bentler, 1999; Kline, 2005) (*X*^*2*^ = 8.653, *df* = 11, *p* = .654, CFI = 1, SRMR = .014, RMSEA [90%-CI] = 0 [0–.027]). The fit of the correlated multidimensional model presents a better fit to the data than the hierarchical model (*X*^*2*^ = 22.792.1, *df* = 14, *p* = .064, CFI = .943, SRMR = .022, RMSEA [90%-CI] = .025 [0–.042]). For the dimension *Identifying Emotions* we observe that the sub-scale *Interpreting Emotional Cues* is loading less strongly, which can be explained by the inherent specificity of this competence. Indeed, compared to the two others sub-scales of this dimension, *Interpreting Emotional Cues* is more oriented towards the identification of emotions in others as opposed to the identification of emotions in oneself. The measurement model and corresponding estimates (standardized factor loading values and latent correlations) are presented in Fig. 2.

#### Stability over Time

It is necessary that assessments remain stable over time (Nunnally & Bernstein, 1967). However, international standards on test development recommend different levels depending on the type of measurement (Chan, 2014).

In our case, stability over time was analyzed on a sub-sample of managers (*N* = 108) who answered the questionnaire a second time after a 6-week interval. The analysis indicates satisfactory indices for the Global EI (GEI) score as well as for the meta-competencies.

The test-retest correlation for GEI was of .73 (*p* < .01), a correlation of .67 (p < .01) was observed for IE, and .55 (p < .01) for UE and .60 (p < .01) for the SME .64 (p < .01). These coefficients are similar to those found in the literature for measures of ability EI, such as the MSCEIT (Mayer-Salovey-Caruso Emotional Intelligence Test) total score stability coefficient at 3-weeks is *r* = .86 (Brackett & Mayer, 2003).

### Criterion Validity

#### Demographic Data

We observe that women have significantly higher scores than men for GEI (*t* = −4089, *df* = 1033; *p* < .001), UE (*t* = −3278, *df* = 1033; p < .001) and SME (*t* = −4,9, *df* = 1033; p < .001). These findings are in line with the results reported in the literature showing that women have greater ability EI than men (Brackett & Mayer, 2003; Ciarrochi et al., 2000; Day & Carroll, 2004; Extremera et al., 2006; Farrelly & Austin, 2007; Goldenberg et al., 2006; John D. Mayer et al., 1999; McIntyre, 2010; Palmer et al., 2005).

In our sample, no significant gender differences have been found for IE (*t* = −0.234, *df* = 1033; *NS*) (Table 5). This result is in line with some studies showing that there are no differences between male and female participants when recognizing facial highly expressive emotions (Fischer et al., 2018; Hoffmann et al., 2008). Indeed, in QEPro the items measuring this meta-component use descriptions corresponding to a moderate/high intensity level of emotional experiences. More research is needed, on how male and female managers exactly differ in their identification of subtle emotion cues.

**Table 5:** ANOVA Results, Mean, Standard Deviation and differences on means and standard error for Male (M; *N* = 535) and Female (F; *N* = 500) managers on QEPro

	Mean (SD)	F	Differences		ANOVA		
	M		d (means)	d (Standard error)	*df*	*F*	p
GEI	0.4 (0.1)	0.43 (0.11)	−0.027	0.007	1/1033	−4.089	0.00
IE	0.47 (0.13)	0.47 (0.14)	−0.002	0.009	1/1033	−0.234	0.82
UE	0.41 (0.22)	0.45 (0.21)	−0.044	0.013	1/1033	−3.278	0.001
SME	0.30 (0.15)	0.35 (0.17)	−0.048	0.010	1/1033	−4.900	0.000

GEI: General Emotional Inteligence; IE: Identifying Emotions; UE: Understanding Emotions; SME: Strategic Management of Emotions

We observe that age is fairly independent from GEI as well as the meta-competencies (Table 6). No relation was found between age and IE (*r* = − .01) and a weak and negative relation (*r* = −.06 to *r* = −.08) was observed with the other abilities and GEI. These results are in line with, on the one hand, studies reporting low negative correlations (Cabello et al., 2014; Day & Carroll, 2004; Palmer et al., 2008) and, on the other hand, with those reporting no relationship between EI and age (Farrelly & Austin, 2007; Webb et al., 2014).

**Table 6:** Correlations between GEI, IE, UE and SME with Age, Initial Training Variables and General Intelligence (*N* = 1035)

	Age	Initial Training	APM-SF
GEI	−0.08*	0.23**	0.28**
IE	−0.01	0.09**	0.15**
UE	−0.08*	0.24**	0.25**
SME	−0.07*	0.11**	0.14**

* p < 0.05; **p < 0.01

GEI: General Emotional Inteligence; IE: Identifying Emotions; UE: Understanding Emotions; SME: Strategic Management of Emotions

**Table 7:** Correlations between GEI, IE, UE and SME with Trait EI measures (EIS; TEIQUE) and Big Five personality dimensions (BFI)

	*N*	GEI	IE	UE	SME
TEIQue	1013	0.03	0.03	−0.01	0.06
EIS	302	−0.04	−0.07	−0.05	0.06
Extraversion (E)	1021	−0.02	0.00	−0.06	0.02
Agreeableness (A)	1021	0.01	0.01	−0.02	0.03
Conscientiousness (C)	1021	−0.03	0.02	−0.09**	0.04
Neuroticism (N)	1021	0.06	−0.01	0.07*	0.06
Openness (O)	1021	0.04	−0.01	0.03	0.06

*p < 0.05; **p < 0.01

GEI: General Emotional Inteligence; IE: Identifying Emotions; UE: Understanding Emotions; SME: Strategic Management of Emotions; TEIQue: Trait Emotional Intelligence Questionnaire; EIS: Emotional Intelligence Scale

With regard to educational levels, we observe a moderate positive correlation between EI and educational levels (ranging from *r* = .09 for IE to *r* = .24 for UE and the GEI). It highlights that education and initial experience are more related than age to EI (GEI, IE, UE & SME) in our sample of adults. This supports the idea that EI develops with experience and education more than as the result of biological ageing alone.

#### General Cognitive Ability

EI and the cognitive ability (APM-SF) present moderate positive correlations ranging from *r* = .14 for IE and SME to *r* = .28 for GEI (Table 6). This suggests some commonalities but mostly that the EI scales do measure a unique, different construct from general intelligence, as measured by the APM-SF. These results are in line with those observed for the MSCEIT (Fabio, 2015, p. 59; *r* = .19 with APM) but differ from those reported by Schlegel and Mortillaro (2019, p. 573) for GECo (*r* = .60 with the Cultural Fair Intelligence Test Scale 2). This questions the relationship between other ability based EI measures and the fluid component of intelligence (Côté, 2010, p. 129).

#### Personality and Trait EI

We observe weak-to-low correlations between ability EI measures (GEI, IE, UE & SME) and the EI trait measures (*r* = −.07 to *r* = .06). These results suggest that QEPro measures a different construct, or at least different aspects of the larger EI construct. This is in line with results reported by Mayer et al. (1999, b) and Brackett and Salovey (2004) concerning the MSCEIT.

We observe low correlations between EI (GEI, IE, UE & SME) and Big Five Personality dimensions as measured by the BFI-FR (*r* = −.09 to *r* = .07), indicating global independence between ability EI and trait personality measures (Matthews et al., 2004). In contrast, we observe moderate-to-high correlations (*r* = −.56 to *r* = .37) between Trait EI measures (TEIQue & EIS) and the Big Five Personality dimensions (BFI-FR). This is in line with results reported by Mikolajczak et al. (2007a, b, c) for TEIQue and BFI. These results suggest that ability EI and trait EI correspond to fundamentally different constructs (Table 8).

**Table 8:** Correlations between the Trait EI measures (EIS and TEIQUE) and the Big Five Dimensions: Extraversion (E), Agreeableness (A), Conscientiousness (C), Neuroticism (N) and Openness (O)

	N	E	A	C	N	O
TEIQue	1013	0.36**	0.37**	0.33**	−0.56**	0.33**
EIS	302	0.24**	0.32**	0.13*	−0.19**	0.37**

*p < 0.05; **p < 0.01

TEIQue: Trait Emotional Intelligence Questionnaire; EIS: Emotional Intelligence Scale

#### Affect Measures

We observe that GEI is negatively related to Alexithymia (correlation for subscales of TAS-20 range from *r* = −.06 to −.16). These results are similar to those of Palmer et al. (2008). Among the subscales of TAS-20 we observe a negative relation between the *Difficulty of Describing Feelings* subscale and UE (*r* = −.09). This suggests that the ability to describe feelings is a central component of understanding emotions. Indeed, the ability to understand emotions partially depends on the ability of the individual to put emotions into words and to describe and understand them (Table 9).

**Table 9:** Correlations between GEI, IE, UE, SME with Affective measures: Alexithymia general. and subscales (TAS-20); Burnout subscales (MBI-GS); Empathy subscales (BES-A); Further Consequences subscale and Immediate Consequence subscale (CFC-14)

	N	GEI	IE	UE	SME
Alexithymia	1023	−0.13**	−0.05	−0,12**	−0.02
Alexithymia: difficulty to describe	1023	−0.10**	−0.03	−0.09**	−0.03
Alexithymia: difficulty to identify	1023	−0.06	−0.05	−0.05	−0.00
Alexithymia: Externally-Oriented thinking	1023	−0.16**	−0.04	−0.16**	−0.13**
Burnout: Exhaustion	302	0.11	0.07	0.05	0.11
Burnout: Cynicism	302	0.07	0.08	−0.01	0.07
Burnout: Loss of professional efficacy	302	−0.08	−0.12*	−0.07	−0.06
Empathy: contagion	302	0.18**	0.14*	0.08	0.13*
Empathy: cognitive	302	0.09	0.03	−0.00	0.16**
Empathy: disconnection	302	−0.13*	−0.04	−0.06	−0.18**
Future consequences	996	0.07*	−0.02	0.07*	0.10**
Immediate consequences	996	−0.07*	−0.03	−0.05	−0.05

*p < 0.05; **p < 0,01

GEI: General Emotional Inteligence; IE: Identifying Emotions; UE: Understanding Emotions; SME: Strategic Management of Emotions

We observe a moderate negative correlation between the subscale *Externally-Oriented Thinking* and UE (*r* = −.16). This indicates that the more externally oriented an individual’s thoughts, the less he/she will be able to understand the emotions. This result suggests that the ability to understand emotions relies partially on the ability to orient one’s thoughts. Similarly, we observe a negative correlation between this subscale and SME (*r* = −.13). This suggests that the more externally oriented an individual’s thoughts, the less he/she will be able to strategically manage emotions. This aspect is of special interest for training and development of EI as it could be interesting to train people to internally orient their thinking in order to develop both abilities: understanding and managing emotions.

Regarding burnout (MBI-GS), we observe a negative correlation between GEI and the *Loss of Efficiency* subscale of Burnout (*r* = −.12). Indeed, part of the burnout is associated with a lack of ability to recognize early signs and indicators of the premises of burnout (Mikolajczak et al., 2007a, b, c). As such, if the identifying emotion step is not precisely performed, understanding and managing emotions cannot be engaged.

We observe interesting patterns between QEPro and Empathy (BES-A). The ability to deeply connect on an emotional level with another person (*Emotional Contagion* subscale) is related to GEI (*r* = .18), IE (*r* = .14) and SME (*r* = .13). UE is less related to the *Emotional Contagion* subscale (r = .08). This indicates that both the ability to identify emotions in self and others as well as the ability to manage emotions prevent from being overpowered and “hijacked” by emotions of others, therefore allowing a deeper emotional connection.

Indeed, empathy is defined as the ability to understand another person’s views and his/her feelings (Rogers, 1951). This definition highlights again the crucial role of IE and SME meta-competencies: to be fully empathic supposes to be able to maintain a certain distance and a distinction between “the self” and “the other,” and not to engage in a complete process of identification (Carré et al., 2013).

For the *Emotional Disconnection* subscale (BES-A) we observe a low negative correlation with SME (*r* = −.18). As this subscale of Empathy is associated with self-protection against ‘extreme unpleasant emotions’ (Batson et al., 1987; Lamm et al., 2007) it is so forth negatively linked to SME which involves, among others, the ability to be receptive to emotional experiences, even unpleasant ones, in order to manage them properly.

Furthermore, the *Orientation Towards Future Consequences* subscale of the CFC-14 was positively related to UE and SME (*r* = .07 and *r* = .10 respectively). This relationship is in line with the definition of these meta-competencies. Indeed, UE and SME help individuals to channel the emotions which arise, whenever one chooses to forego immediate gratification for greater long-term benefits.

## Discussion

In this article we validated a new ability-based measure of EI dedicated to managers. In line with the EI model proposed by Schlegel and Mortillaro (2019), QEPro model is composed of three branches excluding Mayer and Salovey’s *Facilitation of Thought* branch which is problematic (MacCann et al., 2014; Roberts et al., 2008; Schlegel & Mortillaro, 2019). Furthermore, QEPro model integrates fundamental characteristics of emotions and the research on emotional regulation. In line with Gross (2007), we defined the ability to strategically manage emotions as composed of two competencies: the ability to identify and select the appropriate emotional state in a given situation (*Selecting the Target Emotional State*) and the ability to implement the accurate emotion regulation strategy (*Emotion Regulation*) to reach the target emotional state. In our approach, *target emotional state* is defined as the most efficient emotional state to reach a given operational goal (e.g. enhance group creativity, face to face negotiation, decision making process). In order to address critics related to EI ability measures, QEPro’s items and their scoring method were developed based on theory. Additionally, to enhance ecological and face validity for the target population, the vignettes used to assess the Strategic Management of Emotions (SME) dimension were designed within the Situational Judgement Tests framework (SJT).

Different studies were conducted to assess the psychometric qualities of QEPro. Results yielded preliminary support for the validity of QEPro in a sample of French managers and business executives. Results indicated a good fit both at the item and the factor levels. QEPro also showed good stability over time. To assess convergent and divergent validity, we explored the links between GEI, the three meta-competencies (IE, UE, SME) of QEPro and demographic characteristics and psychological measures. Regarding demographic data, age had a minimum effect in this sample of adults, but respondent’s education level was found to influence QEPro results, suggesting that emotional competencies develop more through experience and education than through biological ageing in this sample of adults. Furthermore, QEPro results varied by gender, which suggests that separated norms for men and women would be useful. QEPro correlated in meaningful and theoretically congruent ways with general intelligence, Trait EI measures, the Big Five factors of personality, and the Affect measures used in this study.

### Limitations and Future Research

The validation process being a continuous process (DeVellis, 2011), we aim at gathering more evidence on the validity of QEPro. In this vein, our research agenda includes further validity investigations (a) exploring the predictive validity of QEPro on managerial outcomes, (b) adapting and validating the QEPro to other populations and cultures, (b) measuring the link between QEPro and the toxicity level of managers, (c) assessing the effect of developmental programs based on the QEPro model. We discuss each in turn below.

#### Predictive Validity of QEPro

Studies exploring the link between the seven competencies of QEPro and a range of specific managerial outcomes such as decision-making process, negotiation, team management, conflict management or crisis management should be conducted. To assess the impact of EI on managerial performance qualitative, quantitative, laboratory and biometric studies could be used.

We aim to explore the predictive power of QEPro through field studies in partnership with French companies from different sectors. These companies are currently collecting key managerial outcomes indicators such as subordinate’s satisfaction level, performance beyond expectations, leadership style, subordinate’s attrition, financial performance, number of reported conflicts in the team. Getting access to such data will not only allow to demonstrate the relations between the QEPro subscales and those outcome variables, but also increase the ecological validity of our results.

As a further step, we also aim to conduct laboratory studies exploring the link between the three meta-competences of QEPro and stress as it has been shown that EI has a moderating impact on cortisol response to stress (Mikolajczak et al., 2007a) and may even work as a “stress buffer” (Lea et al., 2019). Such study would allow to explore the predictive validity of QEPro in relation to stress-induced outcomes (e.g. hormonal levels, skin conduction, heart rate) – as stress management is a crucial competence for managers especially during these difficult times (Hagger et al., 2020; Knight, 2020; Serafini et al., 2020).

#### Adaptation of QEPro to Other Populations and Cultures

Even though QEPro was specifically designed for managers, it could be adapted to other populations working in emotionally demanding environments (e.g. sales, military, nursery; Hochschild, 2012) by recontextualizing the vignettes of the Strategic Management of Emotions dimension (Schmitt & Chan, 2006). In order to capture other professional contexts and to facilitate the test takers ability to identify with the described situations, methods such as focus groups, identification of critical incidents (Flanagan, 1954) and expert survey should be used. This would allow to develop context-parallel versions that would be based on the same scoring method, the validation of those new versions would then follow guidelines used for cultural and linguistic test adaptations (Iliescu, 2017).

#### Intercultural Validation of QEPro

Future research on QEPro should investigate its cross-cultural validity on two levels. On the one hand, the robustness of the model can be explored by examining its factorial invariance across cultures, as it has been done for the MSCEIT (Karim & Weisz, 2010).

On the other hand, the universality of QEPro competencies should be examined (Hambleton & Kanjee, 1995). Indeed, according to past cross-cultural studies on emotion recognition (Elfenbein & Ambady, 2002; Jack et al., 2009) and on emotion regulation (Matsumoto et al., 2008), we hypothesize that two of the meta-competencies of QEPro - Identifying Emotion (IE) and Strategic Management of Emotions (SME) - would be more dependent on cultural context. Understanding the intercultural functioning of these dimensions might benefit from a differential item functioning exploration (Borsa et al., 2012).

#### Investigating the Link between QEPro and Managers’ Emotional Toxicity

For the past decade, researchers have been actively studying abusive and toxic behaviors (e.g. aggressiveness, rudeness, manipulativeness) of individuals in the workplace and their effects on psychological health (e.g. depression) and on performance of employees (Forsyth et al., 2012; Krasikova et al., 2013; LeBreton et al., 2018; Spain et al., 2014; Tepper, 2000). Most of these studies have focused on the so-called *Dark Triad* (DT; Adrian Furnham et al., 2013), namely the three dark personality traits of Psychopathy, Machiavellianism, and Narcissism. Studies showed that these three personality traits are aversive (toxic) and distinct, although they share a number of factors in common (Paulhus & Williams, 2002).

Some studies have focused on a particular type of company employee, namely the managers, and showed that possessing DT traits predicts destructive leadership (Forsyth et al., 2012; Krasikova et al., 2013; Spain et al., 2014).

In recent years, on the basis of an emerging academic literature, a debate has risen about the association between EI and the DT (Jauk et al., 2016). Studies tend to show a negative association between EI and some DTs (Miao et al., 2019). Other studies, which are rarer but have received considerable media coverage (Bariso, 2018; Cummins, 2014), have revealed a positive link between EI and some DTs, often focusing on one or more subdimensions of EI, for instance the ability to regulate emotions (Côté et al., 2011; Davis & Nichols, 2016). Such studies conclude by claiming that individuals who are able to regulate their emotions and those of others, will take advantage of this ability to manipulate the other preferring to serve their own interests in certain situations (Côté et al., 2011; Davis & Nichols, 2016).

The vast majority of studies focusing relationships between EI and the DT have focused on investigating the “trait” approach to emotional intelligence (see the literature review in Jauk et al., 2016; although see Côté et al., 2011 for a notable exception). Only a few studies about the DT have used ability EI tests (Jauk et al., 2016; Zhang et al., 2015), and none to our knowledge has done so on a population of real-world business managers. Thus, we propose to explore the relation between QEPro and Psychopathy, Machiavellianism, and Narcissism, using a sample of real-world managers.

#### Assessing the Effect of Developmental Programs Using QEPro

Recent studies revealed the existence of emotional plasticity (Davidson et al., 2000; Kotsou et al., 2011; Lepousez et al., 2015) legitimating the development of training programs based on QEPro.

To assess the effectiveness of QEPro based training programs we propose to follow Kirkpatrick and Kirkpatrick (2016) guidelines. The authors recommend a longitudinal design with a four-stage evaluation system: (a) Reaction assessment: assessment of the emotional reactions and judgments about the usefulness of the training, (b) Learning evaluation: assessment of the competence development at four different points in time (before the training, directly after the training, six months later, and one year later), (c) Behavior assessment: assessment of whether or not a transfer of skills occurred, and (d) Results: assessment of the impact of the training on organizational performance criteria.

Such an assessment would allow to identify the conditions most conducive for the development of the seven emotional competencies measured by QEPro.

To conclude, the present research offers a fine-grained approach to ability measurement of EI in the workplace. QEPro, which offers advantages over existing ability EI measures, may be especially useful in studies and practices aiming to link EI to management. We believe that the future of EI, both academic and practical, lies in the development of such approaches which tried to address common theoretical and psychometric criticisms of EI.

## Data Availability

The data that supports the findings of the study are available from the corresponding author upon reasonable request.

## Declarations

### Potential Harm and Risks (Economic, Emotional, Legal, Physical, Political, Psychological, Social) to Participants

We believe our study involves very minimal or no risk. Participants were informed, as an introduction to data collection, of the length of the questionnaires administered (1.30 h), the aim of the study (to validate a new EI tool dedicated to managers), that no EI scores will be provided once the questionnaires were completed (as it is the construction phase of our tool). One of the authors offered a webinar and a conference (at emlyon) to debrief some initial results of the study with participants who wanted to. Also, a few weeks after the questionnaire was completed, all participants got access to a free video providing some tricks to help them regulate their emotions in management situations.

### Issues with Privacy and Confidentiality

Before filling the questionnaires, participants were informed that their data will be treated confidentially. Names, phone numbers, credit cards numbers, social security numbers were NOT asked. Gender, age, mother tongue, types of diploma, length of Professional experience, the size and activity sector of the company they work for, seniority in their company and seniority at their current position, the number of persons they supervise directly and indirectly, the type and length of skills trainings they followed, if they were/are coached, were asked. The email address the participants provided was only used to send them the URL access to the tips and tricks videos.
